# Frequency characteristics of temporal and spatial concordance among dynamic indices in inattentive and combined subtypes of attention deficit hyperactivity disorder

**DOI:** 10.3389/fnins.2023.1196290

**Published:** 2023-10-19

**Authors:** Ran Chen, Yun Jiao, Jun-Sa Zhu, Xun-Heng Wang

**Affiliations:** ^1^Jiangsu Key Laboratory of Molecular and Functional Imaging, Department of Radiology, Zhongda Hospital, Medical School of Southeast University, Nanjing, China; ^2^Institute of Biomedical Engineering and Instrumentation, Hangzhou Dianzi University, Hangzhou, China

**Keywords:** attention deficit hyperactivity disorder, resting-state fMRI, subtype, frequency dependence, concordance

## Abstract

Numerous voxel-based resting-state functional magnetic resonance imaging (rs-fMRI) measurements have been used to characterize spontaneous brain activity in attention deficit hyperactivity disorder (ADHD). However, the practical distinctions and commonalities among these intrinsic brain activity measures remain to be fully explored, and whether the functional concordance is related to frequency is still unknown. The study included 25 ADHD, combined type (ADHD-C); 26 ADHD, inattentive type (ADHD-I); and 28 typically developing (TD) children. We calculated the voxel-wise (temporal) and volume-wise (spatial) concordance among dynamic rs-fMRI indices in the slow-5 (0.01–0.027 Hz) and slow-4 (0.027–0.073 Hz) frequency bands, respectively. The spatiotemporal concordance within the slow-4 and slow-5 bands among the ADHD-C, ADHD-I, and TD groups was compared. Although the ADHD-C and ADHD-I groups showed similar volume-wise concordance, comparison analysis revealed that compared with ADHD-C patients, ADHD-I patients exhibited decreased voxel-wise concordance in the right median cingulate and paracingulate gyrus (MCC) and right supplementary motor area (SMA) in the slow-5 band. In addition, the voxel-wise concordance was negatively correlated with the diagnostic scores of ADHD subtypes. Our results suggest that functional concordance is frequency dependent, and dynamic concordance analysis based on specific frequency bands may provide a novel approach for investigating the pathophysiological differences among ADHD subtypes.

## 1. Introduction

Attention deficit hyperactivity disorder (ADHD) is a common and persistent neurodevelopmental childhood disorder and is often characterized by inattention, hyperactivity/impulsivity or both. The disorder can seriously affect patients’ social interactions in terms of difficulty concentrating and poor self-control. ADHD is characterized by strong heterogeneity and familial aggregation (Faraone and Larsson, 2019; Tistarelli et al., 2020). The total prevalence of ADHD in children and adolescents in China was 6.26% (Wang et al., 2017). ADHD encompasses three subtypes: the predominantly inattentive subtype (ADHD-I), the hyperactive/impulsive subtype (ADHD-HI) and the combined subtype (ADHD-C), according to the fifth edition of Diagnostic and Statistical Manual of Mental Disorders (DSM-V). Different ADHD subtypes may have different complex characteristics. ADHD-I is associated with dysfunctions in the fronto-parietal and cerebellar systems of task execution control, while ADHD-C is associated with the default network, highlighting issues related to motivation and emotions in the disorder (Fair et al., 2012). Moreover, subgroups also differ in the degree of cognitive impairment (Karalunas and Nigg, 2020), which may be important for elucidating relevant aspects of neurobiological heterogeneity. ADHD studies still present challenges such as subtype symptom instability that manifests with development (Krasner et al., 2018; Luo et al., 2019) and difficulties in identifying neurophysiological markers (Nigg et al., 2005, 2020). Therefore, it is necessary to further search for effective subtype-specific functional neuroimaging biomarkers to address these challenges.

Resting-state functional magnetic resonance imaging (rs-fMRI) is an MRI analysis that measures the blood oxygen level dependent (BOLD) signals of patients in the absence of external stimulation (Biswal et al., 1995; Fox and Raichle, 2007). It is characterized by simple operation and easy completion for patients with mental disorders. To characterize the fluctuating patterns of brain activity, various voxel-based rs-fMRI metrics have been proposed. Regional homogeneity (ReHo) was first proposed to reflect the degree of concordance between an individual voxel and its neighbors during activity (Zang et al., 2004). The amplitude of low frequency fluctuation (ALFF) and fractional ALFF (fALFF) were subsequently proposed to measure the energy level when the time series of voxels were converted to the frequency scale and the fluctuation amplitude after normalization (Zang et al., 2007; Zou et al., 2008). They have been widely applied to ADHD (An et al., 2013; He et al., 2022). Subsequently, the concepts of degree centrality (DC) (Buckner et al., 2009; Zuo et al., 2012) and voxel mirror homotopic connectivity (VMHC) (Zuo et al., 2010) were developed based on functional integration. DC is used to measure the relationship between individual voxels and the global networks, and VMHC measures the functional connection between symmetrical voxels in the two hemispheres. In addition, global signal correlation (GSC) evaluates the functional connectivity between each voxel and the average time course from global signals (Hahamy et al., 2014; Power et al., 2017). These indices have been widely used to characterize the abnormal intrinsic brain activity of ADHD patients (Jiang et al., 2019; Shang et al., 2021; Chen et al., 2022).

Although there are definitional differences among these intrinsic brain activity indices, the practical distinctions and commonalities among these fMRI measures still need to be fully explored in the absence of a comprehensive neurophysiological perspective on the underlying mechanisms of ADHD. Yan et al. proposed a novel concept, dynamic concordance, to comprehensively explore the interdependent relationship among rs-fMRI indices in terms of temporal and spatial dynamics (Yan et al., 2017). While the optimal methodologies are still under exploration (Hindriks et al., 2016), the temporal dynamic perspective of rs-fMRI measures makes it possible to couple different measures together within individuals. Dynamic functional network analysis revealed that spontaneous neuronal activity shows dynamic fluctuations in the resting state (Calhoun et al., 2014; Preti et al., 2017). It also revealed that the fluctuations in dynamic functional connectivity of intrinsic brain activity are consistent with periods of high and low network modularity, which can be used to investigate the spatiotemporal patterns of time-varying functional connection characteristics (Betzel et al., 2016). Yan et al. conducted a temporal dynamic analysis of healthy individuals and revealed the presence of high and low integration states in the brain. The high concordance state is characterized by increased functional connectivity within and between networks, suggesting more general variations in network segregation and integration (Yan et al., 2017). In addition, dynamic concordance has been applied to mental disorders, and the temporal and spatial concordance values were reduced in patients with Alzheimer’s disease and major depressive disorder compared to healthy controls (Zhu et al., 2019; Tian et al., 2022).

Most studies on neural oscillations in ADHD patients have examined the conventional low frequency band (0.01–0.08 Hz). However, when Buzsaki et al. subdivided the full frequency of oscillations into subbands, they found that oscillations within different bands were related to various neurophysiological activities (Buzsáki and Draguhn, 2004). Previous studies on conventional frequency bands have neglected important information contained in each separated frequency band. The mixed neural oscillation can be divided into six frequency bands from slow-6 to slow-1 according to natural logarithmic linear theory (Buzsáki and Draguhn, 2004). Research has found that blood oxygen level-dependent oscillations in gray matter of the brain are mainly concentrated in slow-5 (0.01–0.027 Hz) and slow-4 (0.027–0.073 Hz), with stronger signals observed in cortical structures in slow-5 and subcortical structures in slow-4 (Zuo et al., 2010). Physiological noises such as respiratory and heart rates are recorded at approximately 0.25 Hz and 1 Hz, respectively (Cordes et al., 2001). Compared to other bands, slow-5 and slow-4 contain less physiological information, which can reduce nuisance noise. As a result, many studies have targeted the slow-5 and slow-4 frequency bands (Bastiaansen et al., 2015; Wang et al., 2020; Yang et al., 2020). Systematic fMRI studies of frequency bands have shown that it is crucial to restrict the frequency range for accurate quantification of neuronal activity due to the differences in the strength and distribution of low-frequency oscillations. However, research on the neural mechanisms of ADHD subtypes across frequency subbands is still lacking.

Our study compared the spatiotemporal concordance within the slow-4 and slow-5 bands among the ADHD-C, ADHD-I and typically developing (TD) groups. The frequency and group interaction effect was analyzed to identify the temporal neurophysiological patterns of ADHD subtypes in frequency subbands. Based on previous research findings, we hypothesized that there would be differences in the dynamic functional concordance of intrinsic brain activity among ADHD subtypes. Our study may enable better understanding of the neurophysiological dysfunction patterns in ADHD subtypes.

## 2. Materials and methods

### 2.1. Participants and data acquisition

The data used in this research were obtained from the Peking University dataset in the ADHD-200 Consortium (ADHD Consortium, 2012). The inclusion criteria of this dataset included: (1) no lifetime history of head trauma with loss of consciousness, (2) no history of neurological disease and no diagnosis of either schizophrenia, affective disorder, pervasive development disorder, or substance abuse and (3) full scale Wechsler Intelligen0ce Scale for Chinese Children-Revised (WISCC-R) score of greater than 80. The participants in the dataset are children aged 9–15 years old and they stopped taking psychostimulant medication for at least 48 h before the scan. Our study continued to exclude subjects based on the following criteria. (1) Female participants were excluded because the proportion of female subjects was small, and sex differences could not be examined. (2) Left-handed subjects were excluded to reduce the influence of handedness. (3) Participants with poor image quality or incomplete information were excluded. (4) Participants with excessive head movement (translation >3 mm, rotation angle >3° or Jenkinson mean frame-wise displacement (FD) greater than 0.2) were excluded. (5) Patients with various ADHD subtypes were differentiated and grouped according to the DSM-IV criteria. Since the Peking University dataset does not include patients with the ADHD-HI subtype, this study only included ADHD-C and ADHD-I patients. Then, we matched the ADHD subtypes with healthy controls based on age to reduce intergroup variance. A total of 25 ADHD-C patients, 26 ADHD-I patients and 28 healthy controls were finally included. All participants were assessed with the Computerized Diagnostic Interview Schedule IV (C-DIS-IV) and the Schedule of Affective Disorders and Schizophrenia for Children—Present and Lifetime Version (K-SADS-PL). The ADHD Rating Scale (ADHD-RS) IV was employed to provide dimensional measures of ADHD symptoms. All participants or legal guardians provided written informed consent, and the study was approved by the Research Ethics Review Board of Institute of Mental Health, Peking University.

The functional and anatomical MRI data were obtained with 3 T Siemens Trio scanners. The scan parameters were 2,000 ms for the repetition time (TR) and 30 ms for the echo time (TE). More detailed scanning parameters can be found in http://fcon_1000.projects.nitrc.org/indi/adhd200/.

### 2.2. fMRI data preprocessing

The resting-state data were preprocessed with Data Processing and Analysis for Brain Imaging (DPABI) (Yan et al., 2016), which was based on Statistical Parametric Mapping software (SPM12) (http://www.fil.ion.ucl.ac.uk/spm) and Resting-State fMRI Data Analysis Toolkit V1.8 (REST 1.8) (http://www.restfmri.net) (Song et al., 2011). The first 10 volumes were discarded to allow participants to adapt to the scanning environment and allow the magnetic field to stabilize. The time delay between slices was corrected, and realignment was performed to correct for head motion between time points. Participants with head motion exceeding 3 mm in translational movement, 3° in rotation, or 0.2 in mean Jenkinson FD were excluded. T1 structural images were aligned to the average functional images through a 6 degrees-of-freedom linear transformation. Then, structural images were segmented into gray matter, white matter and cerebrospinal fluid, and nuisance covariates were regressed out. The segmented images were normalized to the Montreal Neurological Institute (MNI) space using the diffeomorphic anatomical registration through the exponentiated Lie algebra (DARTEL) tool. Finally, functional volumes were resampled into 3 mm isotropic voxels.

### 2.3. Calculation of dynamic rs-fMRI indices

We used a sliding window to analyze the temporal dynamic characteristics of rs-fMRI indices. Hamming windows with a length of 32 TR and a step of 4 TR were applied along the time series. Previous studies have shown that dynamic functional connectivity fluctuations can be captured even with window lengths ranging from 30 to 60 s, and different window lengths do not produce significantly different results (Deng et al., 2016; Preti et al., 2017). Moreover, the step size does not significantly affect the variability of fMRI dynamic characteristics, but the empirical value is equal to one-tenth of the window length (Liao et al., 2019). The window length and step size we used were based on previous literature (Lou et al., 2021; Yang et al., 2022). The following dynamic measurements were calculated in each window:

1. ALFF/fALFF: ALFF is defined as the average power spectrum of the time series after Fourier transform in a particular low-frequency band (Zang et al., 2007), and fALFF is the ratio of the power spectrum in the low-frequency band to the entire frequency range (Zou et al., 2008). ALFF reflects the intensity of neural activity by the amplitude of the spectrum, while fALFF represents the relative contribution of a particular oscillation to the entire detectable frequency range. In this study, we used 0.01–0.027 Hz (slow-5) and 0.027–0.073 Hz (slow-4) bandpass filters to calculate ALFF/fALFF, respectively.
2. ReHo: A method for quantifying functional separation using Kendall’s coordination coefficient to calculate time series between a particular voxel and its nearest neighboring voxels (26 voxels) (Zang et al., 2004). ReHo reflects the degree of concordance in regional neural activity.
3. DC: A method of determining functional integration by calculating Pearson’s correlation coefficient between the time series of each voxel and all gray matter voxels. DC was defined as the sum of positive functional connectivity above the threshold of 0.25 (Buckner et al., 2009; Zuo et al., 2012).
4. GSC: Pearson’s correlation coefficients were used to calculate the correlation between each voxel time course and the average time course from global signals (Hahamy et al., 2014). Then, Fisher’s Z-transformation was performed to obtain GSC maps.
5. VMHC: The average T1 mirrored images were first used as a template to transform the functional images to the standard symmetric space. Then, Pearson’s correlation coefficients between the time course of each voxel and the corresponding symmetric hemispheres were calculated to represent the functional connectivity between any pair of symmetric hemispheres (Zuo et al., 2010). Finally, the VMHC maps were Fisher Z-transformed for subsequent analysis.

The fMRI indices mentioned later in the text referred to dynamic measures, namely, dynamic fALFF/ReHo/DC/GSC/VMHC.

### 2.4. Voxel-wise and volume-wise concordance

Kendall’s W coefficient was used to quantify the degree of concordance among five dynamic rs-fMRI indices (fALFF, ReHo, DC, GSC, and VMHC), since this non-parametric statistic has no assumptions of the distribution and is insensitive to differences in scale among these fMRI measures (Yan et al., 2017). Due to the high collinearity between ALFF and fALFF, to avoid artificially exaggerating concordance measurements, only fALFF was utilized in the analysis. It has been reported that fALFF measurement shows less susceptible to nuisance noise and has higher sensitivity and specificity than ALFF (Zou et al., 2008; Yan et al., 2013). We calculated two kinds of dynamic concordance. (1) Voxel-wise concordance (temporal concordance) was defined as Kendall’s W coefficient of the five dynamic fMRI indices across time windows. Each voxel was assigned a concordance value, which formed a global voxel-wise concordance map in each participant (Yan et al., 2017). Then, the map was smoothed with a 4 mm full width at half maximum (FWHM) Gaussian kernel. (2) Volume-wise concordance (spatial concordance) was calculated within each window by computing Kendall’s W coefficient of fMRI indices across all brain voxels. Kendall’s W from all time windows was averaged to obtain one spatial concordance value for each participant (Yan et al., 2017).

### 2.5. Statistical analysis

The demographic information was assessed with SPSS 26.0. The baseline data of the ADHD-C, ADHD-I and TD groups were checked for normality and homogeneity of variance, and the group comparisons were corrected by Bonferroni adjustment for multiple comparisons. The statistical results are shown in Table 1.

**Table 1:** Demographics and clinical characteristics of the participants.

	ADHD-C	ADHD-I	TD	ANOVA^a^	Group comparisons^b^		
					C-I	C-TD	I-TD
*N*	25	26	28	–	–		
Gender (male)	25	26	28	–	–		
Age (years)	11.6 ± 1.7	12.5 ± 1.7	12.1 ± 1.5	0.148	0.154	0.952	0.935
ADHD index	56.7 ± 7.5	46.0 ± 6.4	28.5 ± 5.9	<0.001	<0.001	<0.001	<0.001
Inattentive index	29.6 ± 3.5	27.8 ± 3.2	15.7 ± 3.5	<0.001	0.239	<0.001	<0.001
Hyper/impulsive index	27.2 ± 5.2	18.3 ± 4.4	12.8 ± 3.5	<0.001	<0.001	<0.001	<0.001
Verbal IQ	115.4 ± 17.5	110.5 ± 14.7	118.0 ± 13.7	0.198	0.771	0.989	0.228
Performance IQ	100.3 ± 12.4	95.2 ± 16.6	110.6 ± 15.5	0.001	0.697	0.044	0.001

Data are presented as the mean ± standard deviation. C, ADHD-combined; I, ADHD-inattentive; TD, typically developing.

a *p* values for ANOVA among the three groups.

b *p* values for group differences after Bonferroni multiple comparison correction.

We constructed a full-factorial analysis of variance (ANOVA) model with frequency band (slow-5 and slow-4) as the within-subject factor and group (ADHD-C, ADHD-I and TD) as the between-subject factor in SPM12. The interaction effects of frequency and group on the concordance maps were compared at the voxel-based level. The mask was constructed from the gray matter of 90% of included individuals, and age and FD were included as covariables. Monte Carlo simulations were performed to correct the resulting images, and the cluster threshold was set at a voxel-wise *p* < 0.001 and survival after 5,000 Monte-Carlo simulations. In addition, a *post hoc* analysis was performed for clusters with significant interaction effects. The significant clusters were extracted as regions of interest (ROIs), and simple effect analysis was performed between two frequency bands and among three groups, respectively, in SPSS. The statistical results were corrected by Bonferroni adjustment. Then, we calculated the mean of a given fMRI measure (fALFF, ReHo, DC, GSC, or VMHC) in the ROIs for each time window. In each participant, we calculated the correlations between pairs of the time series of five fMRI indices. These correlations were then averaged across each group of participants to provide an estimation of the dynamic correlation. Finally, Pearson’s partial correlation analysis was conducted to examine the relationships between the concordance indices of the ROIs and clinical scores, with age and FD as covariables.

## 3. Results

### 3.1. Voxel-wise concordance alterations

**Figure 1:**
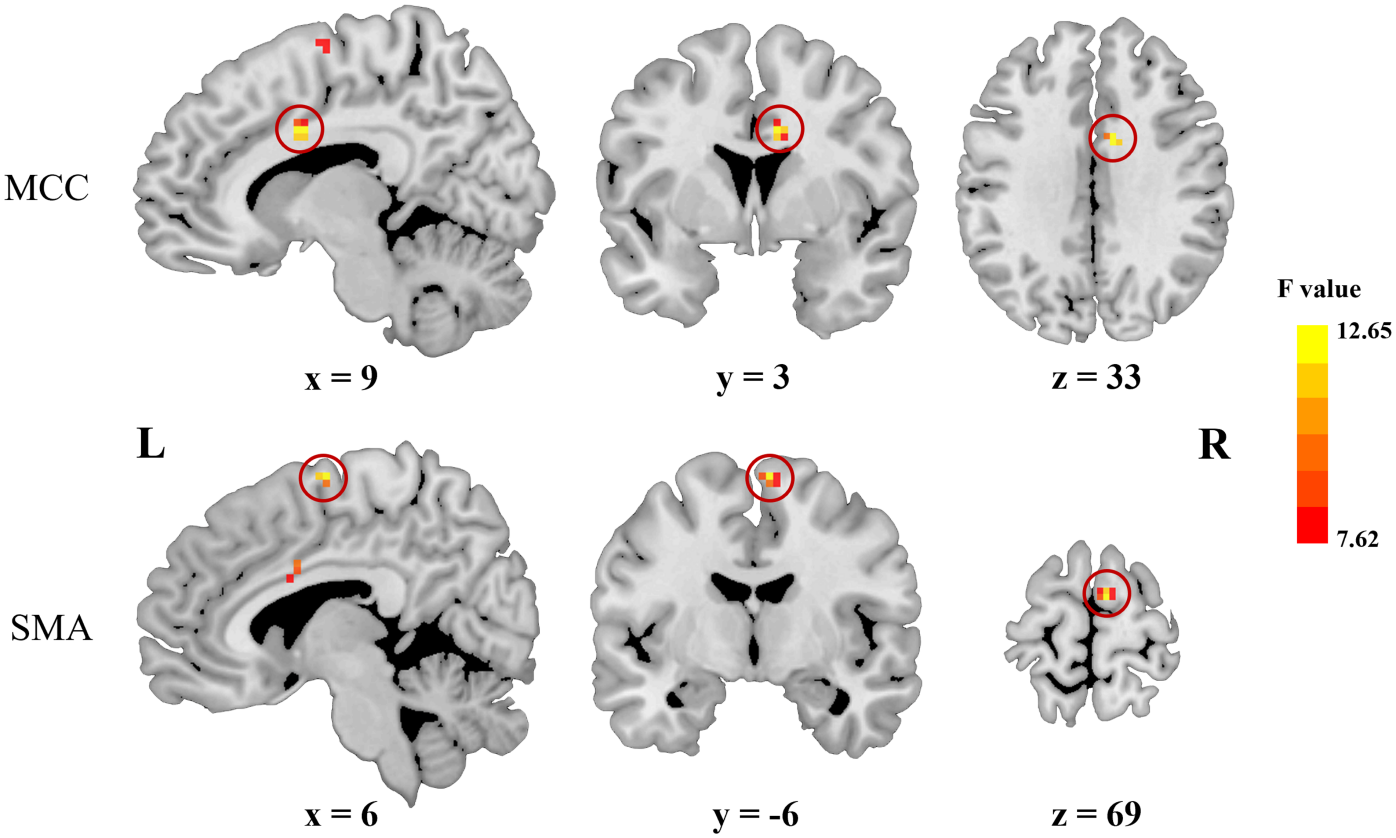
Significant frequency × group interaction effects obtained from ANOVA.

**Figure 2:**
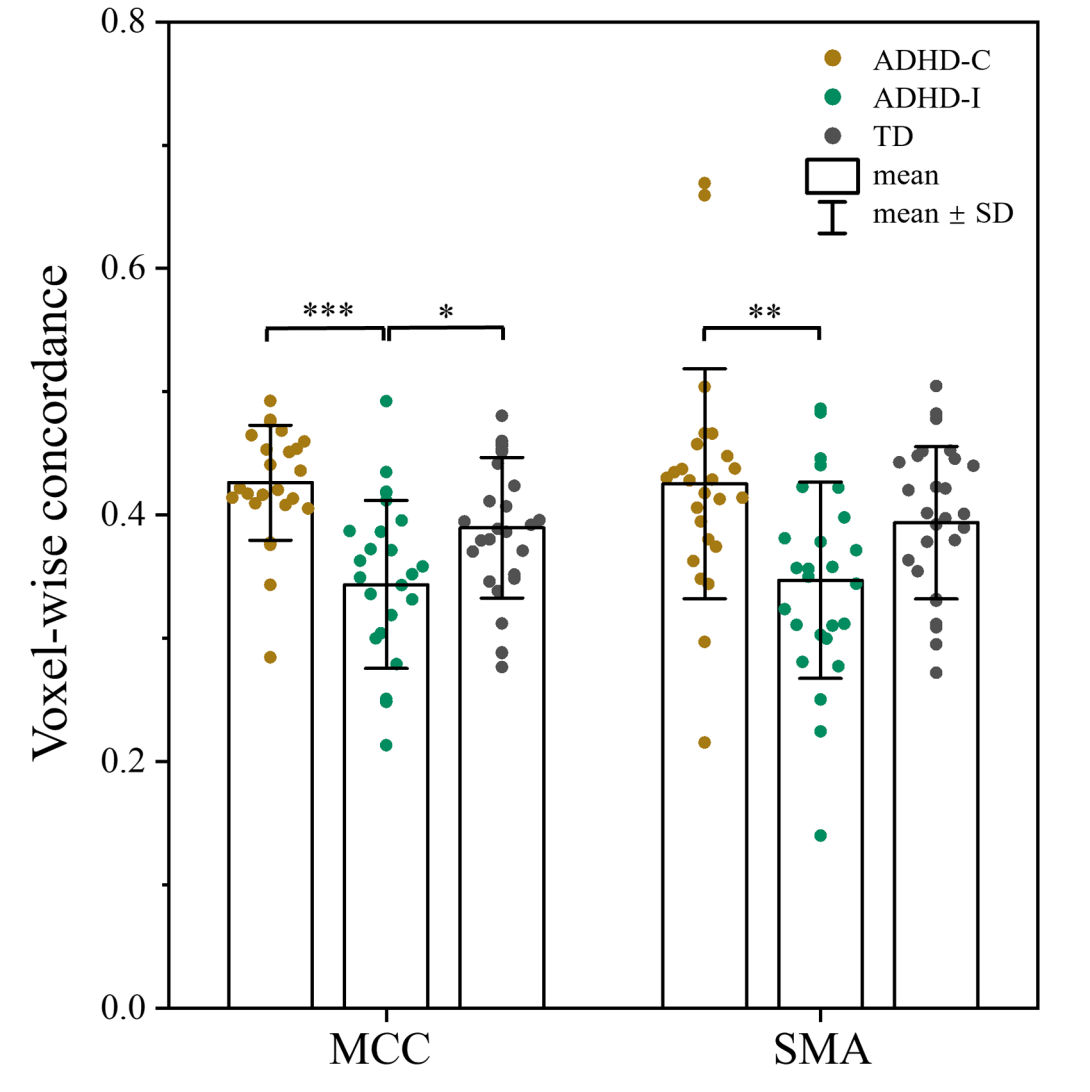
*Post hoc* analysis of voxel-wise concordance among the ADHD-C, ADHD-I, and TD groups in slow-5 with Bonferroni correction. Significant differences are marked by asterisks. *p < 0.05, **p < 0.01, ***p < 0.001.

**Figure 3:**
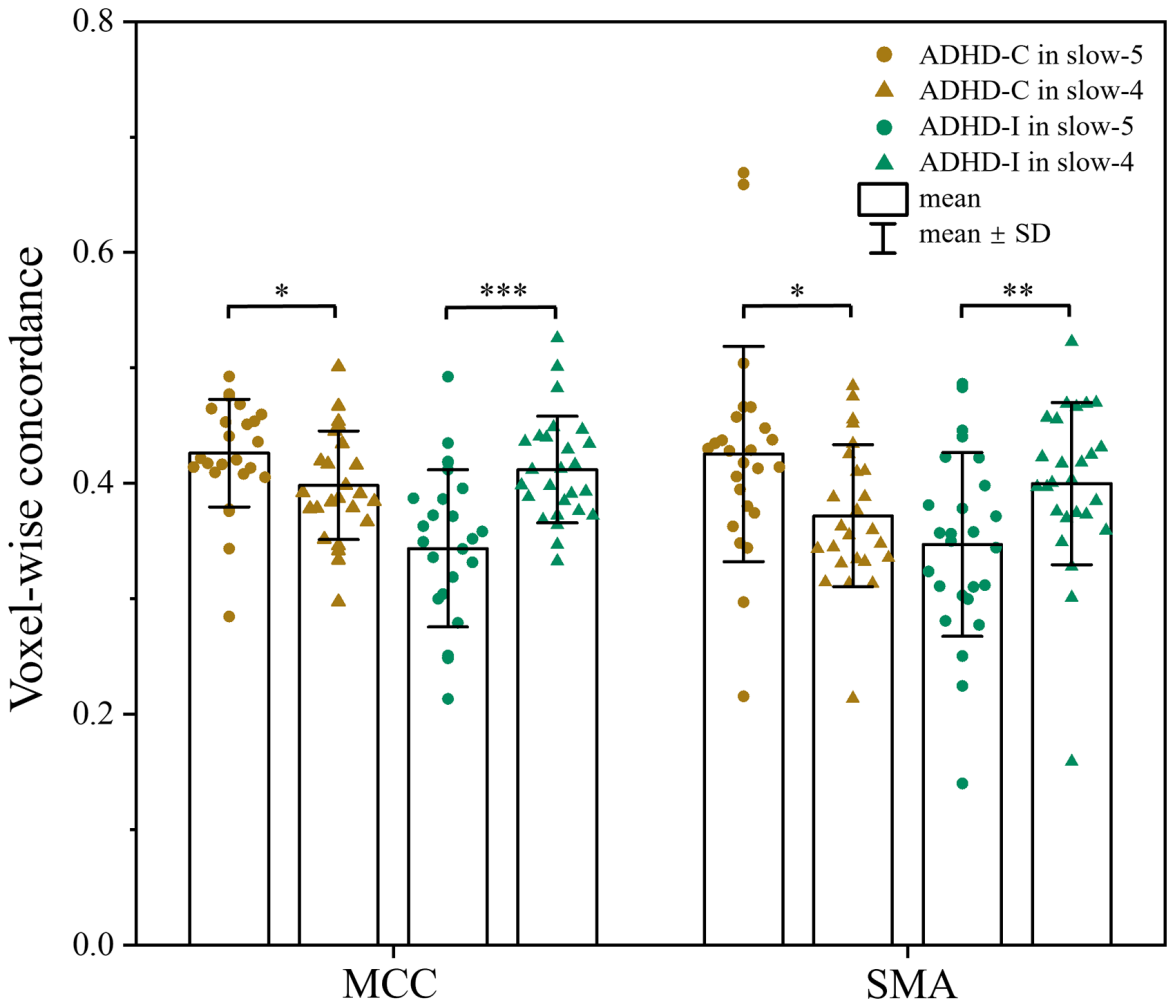
Comparison of voxel-wise concordance in the slow-5 and slow-4 bands. Significant differences are marked by asterisks. *p < 0.05, **p < 0.01, ***p < 0.001.

As shown in Figure 1; Table 2, voxel-wise concordance of the ADHD-C, ADHD-I, and TD groups showed significant differences in the interaction effect in the right median cingulate and paracingulate gyrus (MCC) and right supplementary motor area (SMA). The *post hoc* analysis showed that the between-group differences all occurred in slow-5 (with Bonferroni correction), while no significant differences were found in slow-4 (Figure 2). The ADHD-I group exhibited lower voxel-wise concordance than the ADHD-C group in the MCC and SMA, and the ADHD-I group also showed decreased voxel-wise concordance in the MCC compared with the TD group. The comparison of frequency bands within individuals showed (Figure 3) that both the ADHD-C and ADHD-I groups significantly differed in slow-5 and slow-4. Moreover, compared with other groups, the ADHD-I group showed the lowest voxel concordance in the MCC and SMA in slow-5 (MCC of ADHD-I in slow-5 vs. slow-4, *p* = 0.0006; SMA of ADHD-I in slow-5 vs. slow-4, *p* = 0.003).

**Table 2:** Significant frequency × group interaction effects obtained from ANOVA.

Region	L/R	Peak MNI coordinates			*F*	Cluster size (mm^2^)
		*x*	*y*	*z*		
MCC	R	9	3	33	12.65	324
SMA	R	6	−6	69	12.16	216

MCC, median cingulate and paracingulate gyrus; SMA, supplementary motor area.

### 3.2. Volume-wise concordance alterations

**Figure 4:**
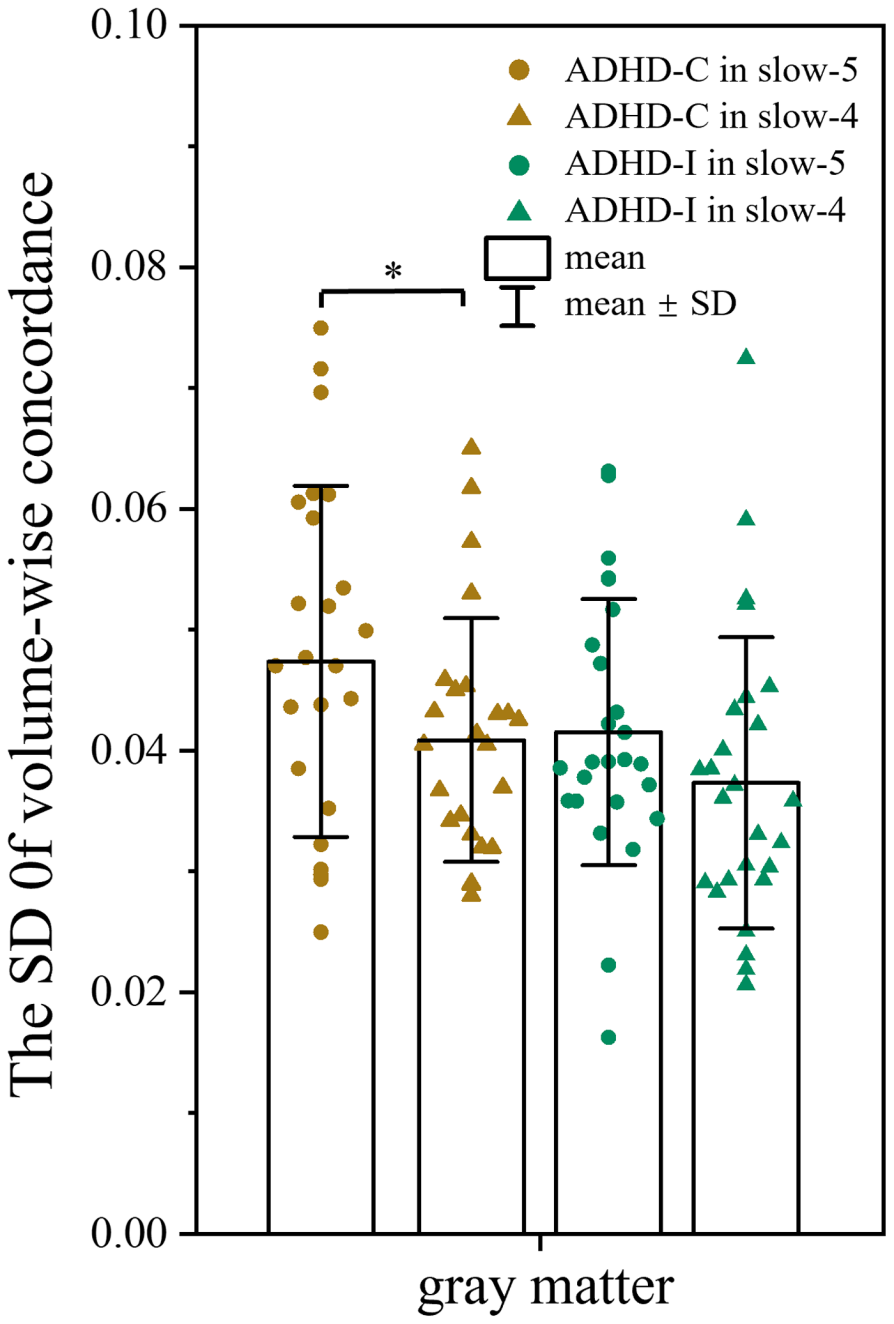
Comparison of the SD of volume-wise concordance in the slow-5 and slow-4 bands. Significant differences are marked by asterisks. *p < 0.05, **p < 0.01, ***p < 0.001.

No significant differences in volume-wise concordance were found among the ADHD-C, ADHD-I, and TD groups. However, the ADHD-C group showed an increased standard deviation (SD) of volume-wise concordance in slow-5 compared with slow-4 (*p* = 0.033) (Figure 4), while the mean value of volume concordance showed no differences (*p* = 0.693).

### 3.3. Correlation among fMRI indices

**Figure 5:**
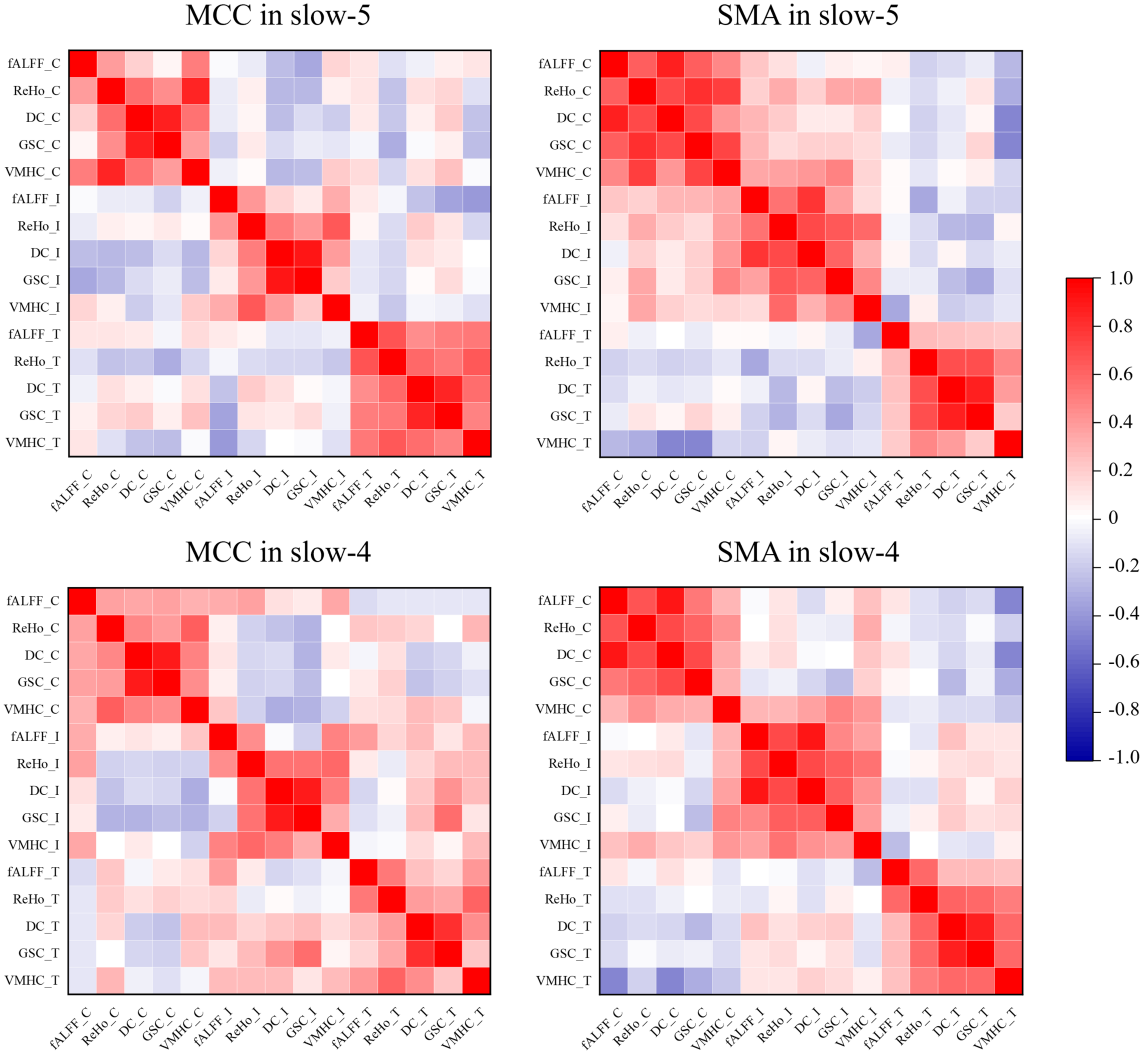
Pearson’s correlations between pairs of dynamic fMRI indices obtained from ROIs. The suffixes C, I, and T indicate the dynamic fMRI measures from the ADHD-C, ADHD-I, and TD groups, respectively.

The ROI-level correlations between pairs of five fMRI indices (fALFF, ReHo, DC, GSC, and VMHC) are shown in Figure 5. We found that the degree of correlation within individuals was higher than that between groups over time. It is worth noting that Yan et al. found a strong global correlation among all measures in healthy individuals (Yan et al., 2017), while we found weak or no correlations between some indices in ROIs for the ADHD-C and ADHD-I groups. The main manifestation was that fALFF was less strongly correlated with GSC and DC.

### 3.4. Correlation with the clinical scores

**Figure 6:**
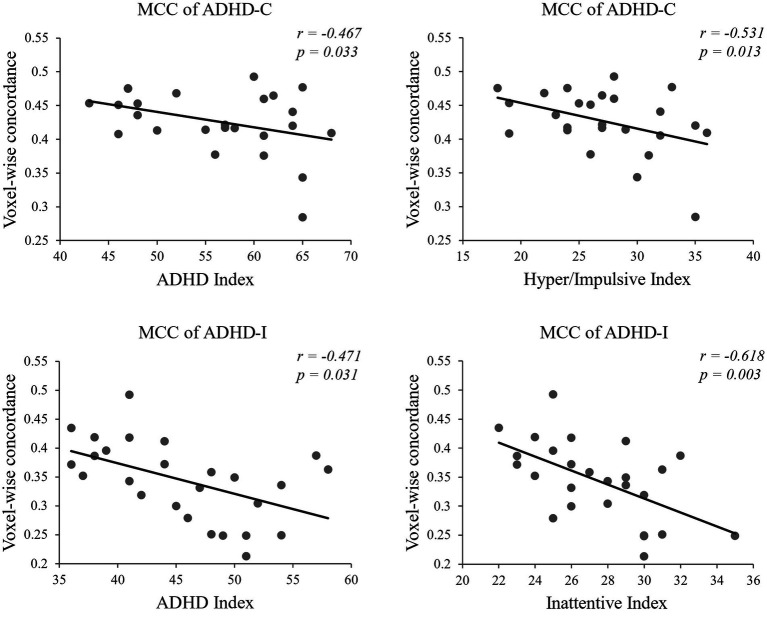
Partial correlation analysis between voxel-wise concordance in slow-5 and clinical scores.

The significant partial correlation analysis results were all in slow-5 (Figure 6). The voxel-wise concordance of the MCC was negatively correlated with ADHD Index and Hyper/Impulsive Index of the ADHD-C group (*r* = −0.467, −0.531; *p* = 0.033, 0.013; uncorrected). The voxel-wise concordance of the MCC was negatively correlated with ADHD Index and Inattentive Index of the ADHD-I group (*r* = −0.471, −0.618; *p* = 0.031, 0.003; uncorrected).

## 4. Discussion

This study compared the temporal and spatial concordance of dynamic rs-fMRI indices among the ADHD-C, ADHD-I and TD groups in the slow-5 and slow-4 bands. The results showed that the voxel-wise concordance of the MCC and SMA was significantly influenced by the interaction of frequency band and group. Moreover, the *post hoc* analysis showed prominent temporal variation among the three groups in the slow-5 band, and the voxel-wise concordance was also correlated with clinical scores. The volume-wise concordance showed no significant group differences, but showed differed between the slow-5 and slow-4 frequency bands.

### 4.1. Variations in temporal concordance

By analyzing the spatiotemporal coupling states of ADHD-C and ADHD-I subtypes, we found that the ADHD-I group exhibited lower temporal concordance than the ADHD-C group in the MCC and SMA. Dynamic concordance states reflect the integrated function of brain network segregation and integration, and are related to functional connectivity within and between networks (Yan et al., 2017). The SMA is often involved in response inhibition and is considered a crucial region for inhibiting unnecessary movements (Mostofsky et al., 2003), planning and executing behavior (Nachev et al., 2008; Simmonds et al., 2008; Rowe et al., 2010) and cognitive functions (Bari and Robbins, 2013). Studies have found decreased connectivity between the SMA and other functional areas in ADHD (Kumar et al., 2022), and disrupted functional activation in the SMA was associated with sustained attention deficits (Fassbender et al., 2015). Moreover, the MCC is an integrated area that operates across functional domains, promoting the selection and motor responses through its connections with the prefrontal cortex and SMA. It plays anatomical and functional integration roles in cognitive control, somatic pain and emotional processing (Shackman et al., 2011; Vogt, 2016; Kragel et al., 2018). Additionally, previous study found that disruptions in the motor response circuit often lead to deficits in response inhibition in children with ADHD (Suskauer et al., 2008).

Dynamic analysis revealed that all fMRI measures were closely coupled with each other in healthy individuals, reflecting common fluctuation patterns underlying the functional aspects (Yan et al., 2017). However, certain fMRI measurement pairs exhibited low correlations within the MCC and SMA of ADHD patients. This suggests a regional impairment of coordination, serving as a possible explanation for the reduced dynamic concordance. Moreover, research has shown that a high concordance state is associated with increased functional connectivity within and between networks (Yan et al., 2017). The decreased voxel-wise concordance in the MCC and SMA may reveal impaired functional connectivity in the motor response areas and disruptions of network integration, which may be related to impulsivity symptoms in ADHD-I patients. It is noteworthy that, compared to the TD group, ADHD-I patients showed a significant decrease in the voxel-wise concordance of MCC, whereas ADHD-C patients did not show the differences. Similar findings have been reported in previous studies. ADHD-I patients showed significant activation deficits in the midline cingulate gyrus and SMA during the oddball-elicited attentional function and they also exhibited decreased responses compared to those with ADHD-C (Orinstein and Stevens, 2014). In terms of brain morphology compared to healthy controls, ADHD-I patients were found to exhibit altered gray matter volume in the cingulate gyrus, while ADHD-C children did not show changes in the cingulate gyrus (Wu et al., 2022). In addition, the dynamic variations in functional connectivity associate with decoding states of wakefulness (Tagliazucchi and Laufs, 2014) and are influenced by alpha and theta oscillation amplitudes in the brain (Chang et al., 2013). ADHD-I patients exhibited reading and decoding impairments related to genetic factors that are not present in ADHD-C (Plourde et al., 2015). The disruption of dynamic coupling in the MCC and SMA over time may help explain differences in coordinated integration when processing potential information between ADHD-C and ADHD-I subtypes (Vogt, 2019). We observed differences in dynamic concordance patterns, which can help explain behavioral differences between ADHD-C and ADHD-I.

### 4.2. Variations in spatial concordance

Spatial concordance did not differ among the three groups, which is consistent with previous studies in ADHD patients (Lou et al., 2021). However, in normal individuals and in populations with other psychiatric disorders, some studies found a decrease in global spatial concordance with increasing age (Yan et al., 2017; Zhu et al., 2018; Tian et al., 2022). The participants in this study were children with a narrow age range, which may account for the lack of significant differences in spatial concordance among the three groups. Temporal concordance was more sensitive than spatial concordance and may be an important tool for detecting abnormal dynamic activity in patients with ADHD subtypes.

### 4.3. Differences between frequency bands

The temporal dynamic concordance significantly differed in the slow-5 frequency band between the ADHD groups, while no significant differences were found in the slow-4 band. The ADHD-C group showed stronger functional concordance in the slow-5 band than in the slow-4 band, while the impaired regions in the ADHD-I group exhibited significantly reduced temporal concordance in slow-5. Although spatial concordance showed no differences among the three groups, there were differences between the slow-5 and slow-4 bands. The increased SD of spatial concordance in slow-5 reflected the increased neuronal activity and fluctuations in the ADHD-I group. The results suggest that frequency subbands may have different pathological importance and that slow-5 is more sensitive to detecting abnormal neural activity among ADHD subtypes than slow-4. Although the sources and meanings of signals from different frequency bands are still unknown, studies have suggested that different subbands are associated with different neural functions and physiological processes (Buzsáki and Draguhn, 2004; Knyazev, 2007). The difference may be caused by different cell structures or specific neuronal processes such as input selection, control binding, plasticity and structural consolidation (Zuo et al., 2010). The high intensity of functional concordance may reflect the overall contribution of neurons (Yan et al., 2017). Moreover, research found that genetic variants of adrenergic receptor genes, which affect sustained attention, were associated with fluctuations in the slow-5 band (Bastiaansen et al., 2015). This may explain the differences in frequency signal sources in ADHD from a genetic marker perspective. The results indicate that both temporal and spatial concordance were frequency dependent, suggesting that sensitive frequency bands should be considered when evaluating intrinsic brain activity in ADHD. However, the results also highlight the specificity and complexity of the brain frequency spectrum, and further research is needed to clarify the relationship between frequency bands and neural activity.

### 4.4. Correlation with the clinical scores

The correlation analysis showed that in the slow-5 band, the voxel-wise concordance of the MCC was negatively correlated with Hyper/Impulsive Index of the ADHD-C group and Inattentive Index of the ADHD-I group. The correlation with the severity of symptoms implies that the functional concordance of the MCC is associated with phenotypic traits. Both the ADHD-C and ADHD-I patients showed an increase in symptom severity and pathological damage with aggravation of neuronal substrate deterioration. Furthermore, since there were no significant differences in global volume-wise concordance among the three groups, no correlation between volume concordance and symptom scores was found. Our results emphasize the ability of functional concordance to capture the neuropathological features of ADHD subtypes and may provide some insights into the dynamic neural activity patterns of ADHD subtypes.

### 4.5. Limitations

The study has some limitations. First, the study had a small sample size, and the results need to be verified in larger datasets. Second, the dataset did not include ADHD-HI patients, so we did not conduct a comparative analysis of the three subtypes of ADHD. Third, the correlation between dynamic concordance and clinical scores did not survive correction for multiple comparison, and no multiple-comparison correction was applied for the correlation analysis. Finally, we did not exclude participants with comorbid oppositional defiant disorder, dysthymia or learning disabilities. More experiments are needed to determine whether these disorders may affect the results.

## 5. Conclusion

The study revealed that ADHD-I and ADHD-C patients showed significant voxel-wise concordance differences in the MCC and SMA. Functional concordance in ADHD patients was frequency dependent and showed greater sensitivity to temporal variations in the slow-5 band than in the slow-4 band. The results suggest that the frequency effects should be considered in future research, and frequency-specific temporal concordance provides new insights into the neurophysiological mechanism of ADHD subtypes.

## Data availability statement

Publicly available datasets were analyzed in this study. This data can be found here: ADHD-200 Sample, 1000 Functional Connectomes Project protocols.

## Ethics statement

The studies involving humans were approved by Research Ethics Review Board of Institute of Mental Health, Peking University. The studies were conducted in accordance with the local legislation and institutional requirements. Written informed consent for participation in this study was provided by the participants’ legal guardians/next of kin.

## Author contributions

RC and YJ contributed to conception and design of the study. RC performed the statistical analysis and wrote the first draft of the manuscript. All authors contributed to the article and approved the submitted version.
