# Corrigendum: Frequency characteristics of temporal and spatial concordance among dynamic indices in inattentive and combined subtypes of attention deficit hyperactivity disorder

**DOI:** 10.3389/fnins.2023.1343392

**Published:** 2023-12-12

**Authors:** Ran Chen, Yun Jiao, Jun-Sa Zhu, Xun-Heng Wang

**Affiliations:** ^1^Jiangsu Key Laboratory of Molecular and Functional Imaging, Department of Radiology, Zhongda Hospital, Medical School of Southeast University, Nanjing, China; ^2^Institute of Biomedical Engineering and Instrumentation, Hangzhou Dianzi University, Hangzhou, China

**Keywords:** attention deficit hyperactivity disorder, resting-state fMRI, subtype, frequency dependence, concordance

In the published article, there was an error in affiliation(s) [1, 2, 3]. Instead of “^1^Jiangsu Key Laboratory of Molecular and Functional Imaging, Medical School of Southeast University, Nanjing, China. ^2^Department of Radiology, Zhongda Hospital, Nanjing, China. ^3^Institute of Biomedical Engineering and Instrumentation, Hangzhou Dianzi University, Hangzhou, China”, it should be “^1^Jiangsu Key Laboratory of Molecular and Functional Imaging, Department of Radiology, Zhongda Hospital, Medical School of Southeast University, Nanjing, China. ^2^Institute of Biomedical Engineering and Instrumentation, Hangzhou Dianzi University, Hangzhou, China. ”

The authors apologize for this error and state that this does not change the scientific conclusions of the article in any way. The original article has been updated.

